# Primary Left Thigh Melanoma Presenting as an Obstructive Hemorrhagic Melanoma of the Small Bowel

**DOI:** 10.7759/cureus.42428

**Published:** 2023-07-25

**Authors:** Lidice Galindo, Catherine Traylor, Larissa Check, Mohamed Faris

**Affiliations:** 1 Internal Medicine, Grand Strand Medical Center, Myrtle Beach, USA

**Keywords:** melanoma, squamous cell carcinoma, basal cell carcinoma, cutaneous melanoma, bowel, melanoma skin cancer, primary melanoma, small bowel melanoma

## Abstract

Primary malignant melanoma of the small intestine is rare and infrequent. However, the small bowel is a relatively common metastatic destination for cutaneous melanoma. Given the fact that primary small intestinal melanoma is a controversial and rare diagnosis, we present a case in which the initial finding suggested a primary tumor. However, the patient was later diagnosed with a small left thigh melanoma after the diagnosis of primary malignant melanoma of the small bowel was established. As a result, we emphasize that all primary small intestinal melanoma must be thoroughly investigated for an alternative primary lesion. Additionally, we question if the diagnosis of primary malignant melanoma of the small bowel needs to be re-classified as small bowel melanoma of unknown primary, especially in cases in which the primary lesion is unidentified.

## Introduction

The existence of a primary small bowel melanoma remains debatable, as a review of the literature suggests there is always the possibility of an unidentified cutaneous lesion. A recent search in scientific literature revealed a total of 43 reported cases of primary small bowel melanoma in comparison to a 2020 report in which only 36 cases had been reported [1]. Small intestinal melanoma commonly presents with symptoms concerning for small bowel obstruction, such as nausea, cramping, and obstipation; however, patients can also present with melena, abdominal distention, intestinal perforation, or diarrhea. The primary treatment is surgical resection of the tumor to improve symptoms and long-term survival. Further workups, such as imaging studies, tumor markers, and histological and immunohistochemical stains of biopsy, are needed to confirm the diagnosis and inform management regarding chemotherapy, immunotherapy, radiation, or surgical resection [2-4]. This case presents a rare presentation of obstructive hemorrhagic metastatic melanoma.

## Case presentation

This case presents a 72-year-old female with a past medical history of type 1 diabetes, a recent stroke on aspirin with an implantable loop recorder, hypothyroidism, hypertension, and hyperlipidemia, who presented to the emergency department complaining of abdominal pain and constipation. The pain was cramping in nature, diffuse throughout the abdomen, and noted to be particularly worse in the left lower quadrant. The patient reported ongoing symptoms for two weeks in addition to a few months of anorexia, early satiety, and weight loss. She reported about two weeks of melena and denied fever and night sweats. She had completed an upper endoscopy and colonoscopy about a year prior to this presentation, which were unremarkable. Interestingly, she had been recently hospitalized for upper extremity hemiparesis and aphasia. During that hospitalization, she was found to have had two subacute strokes and was placed on aspirin 81 mg daily. She denied any other history of cerebrovascular accidents, arrhythmias, myocardial infarctions, hypercoagulable disorders, pregnancy loss, or cancer. She was a non-smoker and not taking any oral contraceptives.

**Figure 1:**
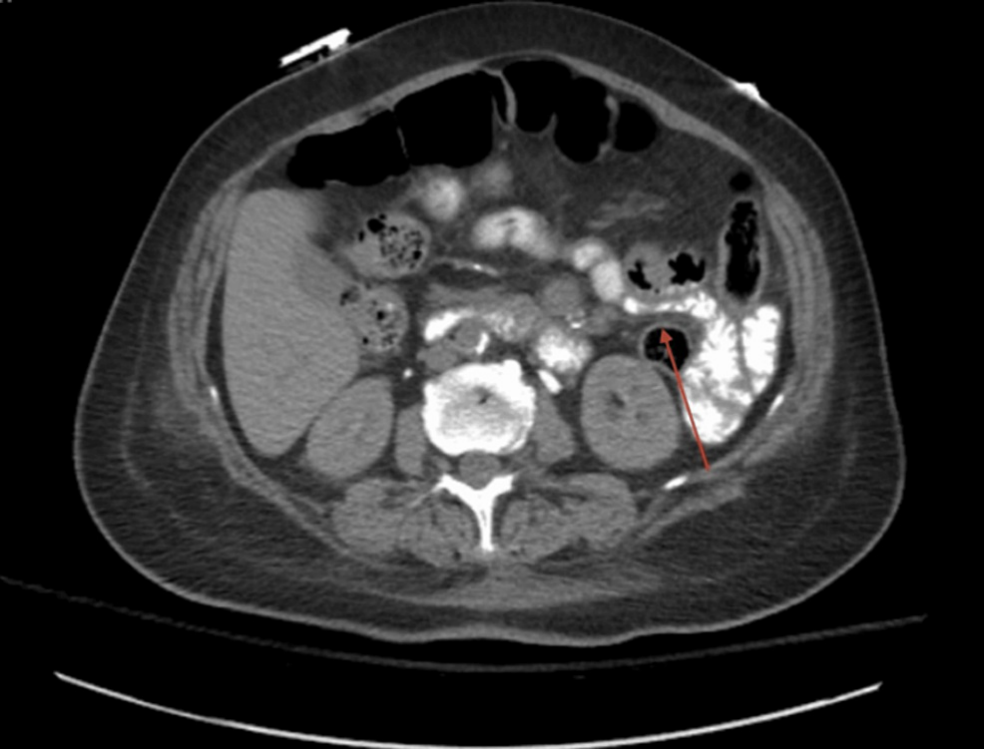
A multi-detector CT imaging of the abdomen and pelvis was performed using the standard protocol bolus administration of oral contrast, which showed abnormal dilated loops of small bowel wall thickening (red arrow).

In the emergency room, she was found to be severely anemic with a hemoglobin of 4.9 (11.6-15.4 gm/dl), hematocrit at 15.0% (34.9-44.1%), slight thrombocytosis with a platelet count of 502 (156-352 K/mm3), and international normalized ratio (INR) was within normal limits. Her comprehensive metabolic panel was unremarkable. Blood glucose was elevated at 181 mg/dL (74-106 mg/dl), and hemoglobin A1c was 6.2% (3.8-5.6%) on an insulin pump. On physical exam, the patient had positive bowel sounds, mild distension, and generalized tenderness. No masses were palpated, and no guarding or rebound was noted. Given the patient’s anorexia and continued abdominal distension, small bowel obstruction was at the top of the differential. She was immediately transfused with a unit of packed red blood cells, stabilized with fluids, and urgently taken for abdominal imaging. A CT of the abdomen and pelvis with oral contrast showed abnormal dilated loops of the small bowel with wall thickening and a poorly defined appearance in the left to right midline lower pelvis, displacing the majority of the large bowel. Proximal to this revealed retained fecal material within the small bowel with a strong consideration of a closed loop obstruction. The wall for the closed loop of the bowel was also abnormally thickened, as shown below in Figure 1.

**Figure 2:**
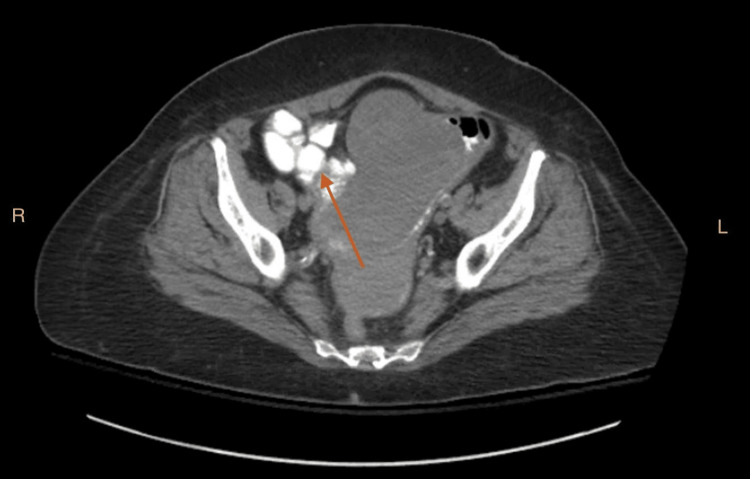
A multi-detector CT imaging of the abdomen and pelvis was performed using the standard protocol bolus administration of oral contrast, which showed a large ill-defined lobular soft tissue mass in the right lower abdomen (red arrow).

The scan further revealed markedly abnormal anatomy with a large, ill-defined, lobular soft tissue mass in the right lower abdominopelvic region measuring up to 11.0 cm x 7.8 cm, best shown in Figure 2.

There was a string sign seen alongside the tumor with contrast detected distally, most likely within the left lower quadrant of the small bowel. Scattered mesenteric lymph nodes without bulky lymphadenopathy were also noted.

Based on these findings, general surgery was immediately consulted. The patient was deemed an appropriate surgical candidate for a small bowel resection as the obstruction was unlikely to self-resolve. An approximately 27.0 cm x 6.0 cm portion of jejunum was resected, and a 12.0 cm x 7.0 cm x 5.5 cm tan-brown necrotic mass abutting the inner intestinal surface was removed. The mass was polycystic; each cyst was filled with hemorrhagic, thin fluid. The solid component of the mass was pink-tan, mottled, and hemorrhagic with areas of necrosis. The mass was found to express BRAF V600E and involve mesenteric tissue. Eighteen lymph nodes were negative for metastasis. Postoperatively, the patient was kept nil per os (NPO) over two days. She was able to slowly advance her diet to clear liquids and finally puréed foods. Upon discharge, it was presumed the patient had a primary malignant melanoma of the small intestine, as no cutaneous lesions were found or noted during her admission.

The patient was evaluated four months later in the primary care office as a follow-up, and per her outpatient oncologist, she had been recently diagnosed with a primary melanoma found on the left thigh, which was characterized as having irregular borders and color variation. This lesion was biopsied and sent for pathological comparison with the small bowel specimen. The left thigh melanoma and small bowel melanoma both expressed BRAF V600E mutation consistent with metastatic melanoma of cutaneous origin.

## Discussion

Melanoma is a tumor arising from the malignant proliferation of melanocytes commonly associated with a high degree of sun exposure leading to DNA mutations [2,3]. Melanocytes are derived from neural crest cells, which can be present on the skin, gastrointestinal tract, and brain. Melanoma is most commonly associated with a BRAF oncogene mutation leading to p.600VE protein substitution. Diagnosis is achieved by punch skin biopsy or excisional biopsy if the lesion is less than 7 mm and not overtly suspicious for malignant melanoma. The most important prognostic factor is the depth of invasion, which determines the survival rate. The most common sites of metastasis are the lungs and gastrointestinal tract (particularly small bowel in malignant melanoma). The prevalence of melanoma in the United States has reached around 50,000 cases per year with an approximate 5% of cases presenting as metastatic at the time of diagnosis [4]. The most common treatment for malignant melanoma is thorough wide, local excision with a focus on palliative surgery, adjuvant immunotherapy including pembrolizumab or nivolumab, and interferon therapy if metastatic [4]. Prevention is aimed at educating patients on decreasing the degree of sun exposure and the use of sunscreen with high SPF (sun protection factor) on exposed skin.

Primary melanoma of the small bowel is rare and the literature review reveals that it is associated with male gender, a predilection for the terminal ileum, and poor prognosis [5]. Diagnostic criteria have been proposed to distinguish primary versus metastatic melanoma, which emphasize the following: the presence of a solitary mucosal lesion in the intestinal epithelium, the absence of melanoma on the skin, and the presence of intramucosal melanocytic lesions in the intestinal epithelium [5,6]. Malignant melanoma is the primary tumor in around 50-60% of cases of metastatic tumors to the small bowel with the majority located in the terminal ileum [7]. Symptoms of melanoma of small bowel can range from chronic abdominal pain, occult or gross bleeding, and weight loss. As in our case, hemorrhagic metastatic melanoma is a rare cause of GI bleeding with patients initially presenting with iron deficiency anemia, as in our patient having a mean corpuscular volume of 71.4 on presentation [7]. In our case, the patient did not meet all criteria of primary melanoma due to the identification of left thigh melanoma on outpatient follow-up.

Diagnosis is made using video capsule endoscopy due to superior specificity and sensitivity compared to CT imaging studies [7]. Surgery is the preferred intervention for diagnosis with a decision to pursue mandatory wide intestinal resection that is dependent on the presence of obstruction, perforation, or severe hemorrhage [6]. In our case, the patient was initially diagnosed with a presumed primary melanoma of the gastrointestinal tract causing obstruction and gastrointestinal bleeding, as evidenced by reported melena prior to presentation and CT abdomen/pelvis findings. Tumor pathology detected BRAF V600E mutation, which was consistent with the metastatic lesion later found on the left thigh. Thus, this finding correlates with the high incidence of metastasis to the gastrointestinal tract in patients with undiagnosed primary cutaneous melanoma. In addition, this case highlights the importance of an initial thorough skin examination if a primary lesion has not been identified and subsequent reclassification as small bowel melanoma of unknown primary.

## Conclusions

Melanoma of the small bowel commonly presents with abdominal pain or obstruction, with the majority of cases originating from metastasis of primary cutaneous melanoma. Various abdominal imaging modalities can be used for diagnosis, including CT and video capsule endoscopy, with final tissue diagnosis obtained via surgical resection commonly demonstrating BRAF oncogene mutation. Ultimately, hemorrhagic and obstructive metastatic melanomas are rare, and this case highlights the importance of a screening skin examination prior to the diagnosis of primary small bowel melanoma.
